# The Impact of Regional Culture on the Psychological Characteristics, Startup Behaviors and Regional Economy of Modern Chinese Business Gangs

**DOI:** 10.3389/fpsyg.2021.732377

**Published:** 2021-12-07

**Authors:** Jun Li, Wanrong Li, Yongchuan Shi

**Affiliations:** ^1^School of Environment and Natural Resources, Zhejiang University of Science and Technology, Hangzhou, China; ^2^School of Accounting, Central University of Finance and Economics, Beijing, China; ^3^College of Innovation and Entrepreneurship, Wenzhou University, Wenzhou, China

**Keywords:** regional cultural differences, psychological characteristics, startup behaviors, modern business gangs, regional economies, new institutional economics

## Abstract

Business gang refers to the enterprise cluster formed by geographical relationship, which has always been the focus of research on entrepreneurship and regional economic development. The research of new institutional economics shows that culture, as an informal system, will change the social psychology, thinking mode and behavior of economic individuals, and provide a good environment for the growth of start-ups, thus affecting economic activities and economic development. Taking the five modern business gangs in China as the research subject, this paper uses the comparative method to analyze the regional cultural differences of the five modern business gangs, as well as the differences of the entrepreneurs’ psychological characteristics and startup behaviors. Through the analysis of the economic data of the provinces where the modern business gangs are located, this paper summarizes the differences of economic development in different regions. It is concluded that regional culture has a significant impact on the development of modern business gangs and regional economy. It is necessary to give full play to the advantages of regional culture and promote the high-quality development of modern business gangs and regional economy.

## Introduction

Underlying each particular economic phenomenon, there are cultural heritage and beliefs, which affect people’s behaviors in economic life, thus affecting economic development (Koslowski, 2010). Taylor, the British anthropologist, defined culture as a complex whole which includes knowledge, belief, customs, and many other capabilities and habits acquired by members of society. Its core is the spread and inheritance of spiritual wealth. The geographic environment, genetic factors and historical development of different countries shape the different regional cultures and thus the culture of the whole nation (Muthukrishna et al., 2020). Culture and economy are closed related, and the economy-culture integration is an inevitable trend worldwide.

The business gang is an enterprise cluster linked by geographical relations. Its long history can be traced back to the top 10 business gangs in the Ming and Qing Dynasties, such as Shanxi gang, Anhui gang, and Longyou gang, which are called Chinese old business gangs. Centered on a particular region and tied by blood and friendship, these old business gangs developed quickly. With the economic transformation, especially the development of regional economy, China has gradually formed five major business gangs in the new era, including Shandong business gang, Southern Jiangsu business gang, Zhejiang business gang, Southern Fujian business gang, and Pearl River Delta business gang. Modern business gangs still have strong regionality. However, in its development process, we can find that many factors affect the formation of the distinctive characteristics of modern business gangs, including geographical location, history and culture, policy system and traffic conditions (Dong and Glaister, 2007).

In the existing research, most scholars explain the reasons for economic development from the aspects of capital, technology, market and labor force. However, few people explore the impact of non-economic factors such as culture on regional economic development, especially from the perspective of psychology. The influence of culture on startup behavior and regional economic development remains unclear. Investigating the impact of regional cultural differences on the development of Chinese modern business gangs is of great significance to the high-quality development of business gang enterprises and regional economy.

## Literature Review

### Modern Business Gangs in China

Since the reform and opening up, three economic zones have emerged in China: Surrounding Bohai Economic Zone, Yangtze River Delta Economic Zone and Pearl River Delta Economic Zone. They can, to a great extent, represent the economic development of China (Lv and You, 2014). With the development of regional economy, the groups of businessmen which can be identified by regional features have evolved. These businessmen groups can be grouped from north to south as Shandong business gang, Southern Jiangsu business gang, Zhejiang business gang, Southern Fujian business gang and Pearl River Delta business gang, referred to as the five modern business gangs in China.

### Regional Cultures in China

Regional culture is created and fostered in the growth of special groups. It not only inherits the Chinese traditional culture, but also constitutes the mainstream culture of economic globalization and the culture of global entrepreneurship and innovation. Modern business gangs inherit the essence of traditional culture. One particular culture is inseparable from the continuity of local culture. Such continuity will produce different folk culture, and thus a different regional culture. One of the key characteristics of regional culture is that its inherent tendency to traditional culture creates various regional cultures with distinguishing characteristics (Nong, 2016). The regional cultures in China are characterized by diversity, openness, compatibility, innovation, friendship and kinship, such as Qilu culture, Wuyue culture and Southern Fujian culture.

### Relationship Between Culture and Economy

Research on the relationship between culture and economic development can be traced back to Adam Smith, the father of economics. He noted that any market economy can only function properly on the basis of shared morality, i.e., adherence to contracts, payment commitments and respect for market partners. In other words, specific cultural concepts are essential for market expansion and economic development (Yerznkyan and Gassner, 2018). Max Weber was among the first to regard culture as a source of endogenous behavior patterns, and he believed that the “spirit of capitalism” from religious ethics, being a unique value system in modern Europe, drove the reform of people’s behavior and eventually promoted economic performance (Isard, 1949).

Krugman examined the impact of culture, ideas and institutions on economic development and economic structure, especially the impact of labor market and labor practices on the social acceptance of labor organization, labor control, forced labor and also its changes. He concluded that different societies had different labor supply systems, which reflected cultural backgrounds and beliefs, while the culture would, in turn, influence economic behavior (Krugman, 1991).

Development economists also value the role of culture in economic development, and some of them have examined the relationship between culture and economic development from different perspectives (Prasetyo et al., 2020). Schumpeter investigated the constraints of culture on innovation, i.e., its impact on economic development (Baldwin, 1999). Boston University has established the Institute for the Study of Economic Culture, specializing in the relationship between social-economic change and culture, as well as economic culture research around the world. Peter L. Berger, director of the Institute, holds that there is a crucial link between culture and economy, and culture has different impact on the economy at different stages of economic development (Baldwin et al., 2001).

Any economic system has its own moral basis or value significance, and economic development requires certain political, cultural and moral conditions (Yu and Nan, 2013). Cultural factors can provide values which will directly affect people’s beliefs, and its social function is mainly to provide conventional code of conduct for economic and political production (Zhang, 2010).

From the current literature, the research on culture and economic development is still in the exploratory stage, and most discussions revolve around the relationship between culture and economic development, that is, the role of culture on economic development and the impact of economy on culture. New institutional economics takes the essence and behavior of “individual” as the logical starting point of the economic theoretical system, pays attention to the study of the relationship between man, system and economic activities, and believes that system plays a decisive role in economic development. At present, there are few studies on the relationship between culture and economy from the perspective of the psychological characteristics and entrepreneurial behavior of business gangs. This paper focuses on the “individual” in the new institutional economics, and believes that the distinctive regional culture plays a potential role in the formation of entrepreneurship. The psychological and behavioral characteristics of entrepreneurs will have an impact on the business model of enterprises and regional economy. Therefore, this paper has unique theoretical research value.

### Relationship Between Regional Culture and Regional Economy

Regional culture refers to the culture with obvious local characteristics formed under different geographical environment and natural conditions (Quek and Storm, 2012). Exerting a profound influence on the development of regional economy, regional culture on one hand provides intellectual support and spiritual impetus for the development of regional economy, while on the other hand it can hinder the development of regional economy because its conservatism would refuse to learn from more advanced cultures (Wang, 2018).

Xia (2000) analyzed the impact of regional culture on regional economic development and concluded that regional culture had an important impact on regional economic development, regional cultural stereotype would restrict the communication and exchanges among regional economies and technologies, and the renewal of cultural values had promoted the transformation of regional economic development model. Examining the model of economic development in Zhejiang Province, Zhang and Tai (2015) suggested that the economic development in Zhejiang correlated with its innovative culture. This culture promoted technological and institutional innovation, which in turn influenced economic development. Focusing on the economic development in Zhejiang Province, Wu and Gao (2007) analyzed the connection among culture, entrepreneurship and economic development. Chen and Han (2008) investigated the impact of the culture on economic development from three aspects, and suggested that cultural factors influenced economic development through the choice of entrepreneurs, intellectual assets and economic structure. Regional economy can determine and strengthen the development patterns, structures, types and characteristics of regional culture, and meanwhile provide regional culture with material conditions (Chen and Han, 2008). Regional culture can promote or hinder the development of regional economy. When regional culture is integrated into regional economy, it can help to form an economic model with local characteristics.

Overall, more and more scholars begin to pay attention to the role of culture on economic development. However, previous studies on regional economic development from the perspective of regional culture failed to build a complete system. The permeability of regional culture imperceptibly affects the main body of regional development. By affecting entrepreneurs’ psychological characteristics and entrepreneurial behavior, enterprises show regional characteristics in participating in regional economic development. Business gang is an enterprise cluster formed by geographical relations. It is deeply influenced by regional culture, and regional economy is closely related to the development of Business gangs. This paper will take China’s modern business gangs as the research subject, explore the impact of regional culture on entrepreneurs, business gangs and regional economic development, and put forward relevant countermeasures and suggestions for the development of business gangs and regional economy. Thus this research has certain practical significance.

## Theory and Hypotheses

### New Institutional Economics

In the study of the relationship between culture and regional economic growth, new institutional economics has provided an economic explanation for the role of culture on economic growth. Based on in-depth analysis of historical data, Douglas North introduced culture into the research in economic and institutional change and economic performance. North believes that culture, as an indispensable variable that influences and constrains the enforcement, ultimately determines economic growth, and that culture not only plays the role of shaping formal rules, but also supports the informal constraints, which is a component of institutions. He stresses that the purpose of institutional economics is to study how people make decisions in the real world and how they change the world in the context of institutional evolution (North, 1955).

North’s definition of institutions consists of both formal and informal constraints. He notes that institutions have been devised by human beings to create order and reduce uncertainty in exchange. The so-called formal institutions are the norms and constraints that members of a society or organization have consciously developed through a certain procedure and require them to follow, such as the political and legal rules. Informal institutions include conventions, customs, cultural heritage, beliefs and ideology that people have gradually formed in social life, which will informally constrain human behaviors. According to North (1955), informal constrains come from the cultural heritage of value, from the extension and application of formal rules to exchanges, and from attempts to solve problems in collaboration.

There are two important representatives in the theoretical research on new economic institutionalism. Galbraith, an American economist, advocates that social psychology, behavioral motivation and mode of thinking should be regarded as the performance of “system,” and its impact on the economy should be paid attention to. In terms of research methods, he opposes the simple quantitative analysis method and advocates comprehensive comparative analysis, which will fully demonstrate the problem. British economist Coase who believes that all economic phenomena should be explained in essence according to individual nature, endowment, goals and beliefs, and culture and system have different degrees of influence on individual thinking mode and behavior mode, that is, the analysis of actual human behavior can better explain and describe human nature and human economic activities.

### Hypotheses

From the perspective of new economic institutionalism, an individual is first of all a “social person” and “organizational person,” not an “economic person.” Human behavior depends largely on his social and cultural environment. Dynamic psychology (Woodworth, 1916) believes that any human behavior has a reason rather than accidental spontaneity. Psychology should answer how and why human behavior occurs based on human behavior in activities and human internal psychological process. Planned behavior theory (Ajzen, 1991) holds that individual and social and cultural factors (such as personality, intelligence, experience, age, gender, cultural background, etc.) indirectly affect behavior attitude, subjective norms and perceived behavior control, and finally affect behavior intention and behavior. Marshall (1890) noted that cultural factors (including religion, morality, ideas and ideals) and economic motivation also determine people’s behaviors. Different regional cultures have their own characteristics and have a subtle impact on individuals. Specifically, different business entrepreneurs studied in this paper will form different psychological characteristics and behavior styles. Thus, our first hypothesis is presented as follows:

H1. Regional culture will affect the entrepreneurs’ psychological characteristics and entrepreneurial behaviors.

New institutional economics takes individual’s essence and behavior as the logical starting point of the economic theoretical system. New institutional economics pays attention to the study of the relationship between man, system and economic activities, and believes that system plays a decisive role in economic development. All economic activities are carried out by people, and economic development is determined by people’s economic activities and behaviors. The establishment and development of enterprise organizations is also based on human activities, while human activities, behaviors and logic are determined by human psychology and motivation, which are affected in turn by the system in their life. By influencing entrepreneurs’ psychological characteristics and entrepreneurial behaviors, regional culture makes enterprises present different industrial choices and business strategies in participating in regional economic development. Therefore, we develop our second hypothesis below:

H2. It is believed that entrepreneurs’ psychological characteristics and entrepreneurial behaviors will affect the development of enterprises and business gangs.

The new institutional school, represented by Hayek and Douglas North, believe that modern economic growth is inseparable from cultural change and explores the cultural factors behind economic growth. Culture, as an important part of “informal system,” has been incorporated into the economic system. Development economists also recognize that culture has an impact on economic development, but they fail to explain the relationship between regional culture and economic development in economic development. Based on the above theories, our third hypothesis is presented as follows:

H3. Enterprises and business gangs affected by different regional cultures will have a certain impact on regional economic development.

**FIGURE 1:**
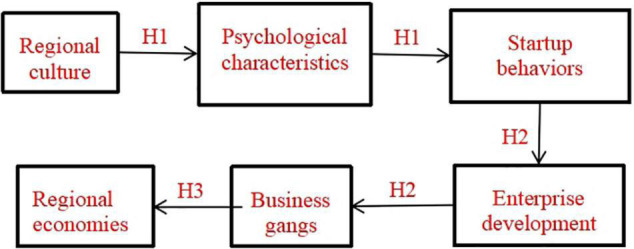
Hypothetical model for the impact of regional cultural differences on modern business gangs.

To sum up, based on the theory of new institutional economics, this paper puts forward the following assumptions: regional culture, as a kind of informal system, facilitates the economic subjects to form different psychological characteristics, resulting in the differences in entrepreneurs’ motivation and thinking mode for specific startup behaviors, so as to affect the business philosophy and development model. This will form the common characteristics of business gangs in a particular region and have an important impact on regional economic development (see Figure 1).

## Methods

This paper uses the comparative method to explore the impact of regional culture on the economic development of modern business gangs. Comparative method can be understood as a method of investigating two or more related things, looking for their similarities and differences, and exploring general laws and special laws according to certain standards.

Therefore, this paper summarizes the differences of regional cultures through literature review. At the same time, this paper also compares the influence of regional culture on the behavior characteristics of the five modern business gangs. By sorting out the relevant data in the National Statistical Yearbook, this paper analyzes and compares the economic situation of the provinces where China’s five modern business gangs are located. Based on these comparisons, we attempt to verify the hypothesis and put forward some suggestions.

### Comparative Analysis of Regional Cultures

#### Culture of Shandong Businessmen

With a strong Qi-lu cultural identity, Shandong business gang displays the pragmatism of northerners and also the astuteness of southerners. However, they have close ties with politics and the government (Ge, 2008). Shandong business gang run the business with traditional Chinese culture. Greatly influenced by Confucian culture, they firmly believe the credos of manners, righteousness, benevolence, wisdom and credit. Meanwhile, they value and know well *The Art of War*. So they are said to have “virtue and morality” on left hand and a sword on right hand (Gao, 2012). According to the culture pedigree, Shandong is at the north of the Huaihe River, but geographically it belongs to east China. As the descendants of Sun-zi and Lao-zi, Shandong businessmen have surprisingly kept a balance between *The Art of War* and *Tao and The* (Chen, 2004).

#### Culture of Jiangsu Businessmen

With a strong Wu cultural identity, southern Jiangsu businessmen value balance, collectivity and hierarchy. They do not have a strong personality. In addition, southern Jiangsu culture is typical of water culture, which, to a large extent, forms the flexibility and modesty of Jiangsu people. In such a historical and cultural context and within the economic radiation of Shanghai, entrepreneurs here are careful and good at management. The spirit passed down by southern Jiangsu business gang values three things. One is innovative and enterprising spirit. The hard-working and strong Jiangsu businessmen set up business and factories and develop trade industry. Second is team spirit. They strive for mutual benefits and win-win results in business cooperation and competition. Third, the quality of honesty and pragmatism. Known for their high literacy, Jiangsu businessmen are ambitious but also realistic and practical in doing business.

#### Culture of Zhejiang Businessmen

Zhejiang businessmen have a deep imprint of eastern Zhejiang culture, and their cultural foundation is Yongjia culture, which values practicability and emphasizes individuality and capacity. In their career development, they always have a sense of leadership and entrepreneurship, while in personal development, they have a strong self-awareness. They not only attach importance to their own development, but also the development of group. Zhejiang businessmen are hard-working and enterprising (Zhejiang Business website, 2006). The culture of Zhejiang businessmen includes the system of beliefs and values created by Zhejiang businessmen, which is deeply rooted in their startup behaviors and is the whole of their beliefs, emotions, values, code of conducts and moral rules (Feng et al., 2009).

#### Culture of Fujian Businessmen

Fujian businessmen are the typical representative of China’s marine civilization, and their most important characteristic is international- and market-oriented. In the culture of Fujian businessmen, credit is a promise and mortgage for the future. The symbiosis of information, trust and reputation is the righteousness. The “compatibility of righteousness and benefit” has been believed by Fujian businessmen for generations. The internal logic of integrity first is that “the brand is priceless,” which is one of the most valuable experiences brought back by Fujian businessmen after participating in the competition in Southeast Asia and even the global market. In addition to brand awareness, they believe that the market economy also needs contract awareness, which is the basis of modern civilization. For enterprises, survival, development, operation and management are also based on contract. Credit, brand and contract spirit constitute the cultural factors of contemporary Fujian businessmen.

#### Culture of Guangdong Businessmen

Guangdong business culture is mainly composed of three parts: smart and open Guangfu culture, hardworking Hakka culture, and capable and competent Chaoshan culture. Taking advantage of the reform and special economic zones, the business gang in the Pearl River Delta have learned from businessmen in Hong Kong and Taiwan. They are bold in doing business and have the spirit of freedom, openness, adventure, exploration, pragmatism and innovation. The Guangdong businessmen have the excellent quality of hardworking. Through their unremitting efforts, they have produced a southern Guangdong civilization and Guangdong merchant culture with local characteristics. Especially since China’s reform and opening up in the 1980s, Guangdong businessmen have quickly become the leader of the national economy with special geographical and policy advantages.

### Comparison of the Impacts of Regional Culture on the Psychological Characteristics and Startup Behaviors of Modern Business Gangs

#### The Influence of Qi-lu Culture on the Development of Enterprises in Shandong

As the birthplace of Confucian culture, Qi-lu culture have influenced generations of Shandong people. Shandong businessmen have a profound mark of Confucian culture. Influenced by the Confucian idea of “morality and righteousness first,” Shandong businessmen attach importance to righteousness over profit in doing business and stress business ethics and business quality. Confucian culture has shaped the elegant character of Shandong people. The most important way of “self-cultivation” is to worship benevolence and etiquette, which is reflected in the business philosophy as customer first. Businessmen and customers follow the principles of “benevolence” and “courtesy,” just like Ruifuxiang, a century old enterprise, whose attitude toward customers is always modest, polite and smiling. Founded in Jinan in 1862, Ruifuxiang has won many awards, such as “China’s time-honored brand,” “the first brand of Chinese silk,” “intangible cultural heritage,” and “famous brand trusted by Chinese consumers.”

In addition, the Confucian thought of “self-restraint” is reflected by the businessmen’s personal qualities such as being rigorous, serious and hardworking. For example, Haier Group, founded in Qingdao in 1984, has always adhered to the innovation system of user-demand-centered to drive the sustainable and healthy development of the enterprise, and has now become one of the world’s largest household appliance manufacturers. Lushang group, a large state-owned enterprise established by Shandong Provincial Department of Commerce in 1992, has grown into a large enterprise group with modern retail as its main industry and biopharmaceutical and real estate as its key industries after nearly 30 years of development. The enterprise has gradually accumulated and precipitated the enterprise spirit, philosophy and values of “hard working, self-reliance, cooperation and dedication” with the characteristics of Shandong businessmen.

#### The Influence of Southern Jiangsu Business Culture on the Development of Enterprises in Jiangsu

Entrepreneurs in Jiangsu are generally hardworking, thrifty, and practical. Since the region is densely populated, everyone is good at planning and management. In terms of the type of entrepreneurs, there are more managerial and political entrepreneurs in Jiangsu, rather than strategic and technical entrepreneurs as in other regions. As the saying goes, “Advisers from Shaoxing, and stewards from Suzhou.” The rise and development of Southern Jiangsu businessmen is particularly prominent in building enterprises. For example, the representatives of Southern Jiangsu businessmen Zhang Jian and Rong Brothers set up factories in Nantong and revitalized industry and education, making Jiangsu the birthplace of China’s national industry and the Pioneer Area of modern education. The four famous industrial and commercial masters of “Rong, Xue, Tang, and Yang” in Wuxi have successively established yarn mills, silk mills, flour mills and brick and tile factories, which has laid a foundation for the development of China’s industrial and commercial economy.

Under the influence of the Southern Jiangsu business culture, the Southern Jiangsu business gang has paid attention to the effective organization and overall operation. In 1902, Suzhou businessmen established the southern Jiangsu General Chamber of Commerce to actively promote local products and revitalize the local industrial and commercial economy. Shen Laizhou, a Jiangsu businessman, started from a small shop and built “Hengyuanxiang” into a “wool kingdom” in China, which is still famous today. Suning Holding Group, founded in 1990, is a company focusing on industrial investment, equity investment and asset management. Among the top 100 enterprises in Jiangsu Province in 2020, Suning Holding Group continued to lead with an annual operating revenue of 282.94 billion yuan, and took the initiative to adapt to the in-depth reform of the retail industry, an increase of 3.13 billion yuan over the previous year, showing a strong potential for development.

#### The Influence of Zhejiang Business Culture on the Development of Enterprises in Zhejiang

The spirit of Zhejiang businessmen includes the entrepreneurial spirit, open spirit, the spirit of calling for change and the spirit of honesty. Modern Zhejiang businessmen do everything possible to improve their brands, expand the market, make independent innovation and improve management. They are good at creating opportunities where there are no opportunities, seize opportunities where there are opportunities, and create a number of distinctive industrial clusters and professional markets. For example, Tongxiang does not produce wool, but has the largest sweater market in China; Yuyao does not produce plastics, but has the largest plastic market in China; Haining does not produce leather, but has the largest leather market in China; Jiashan has no forest, but has the largest wood processing market in China. Thus, Zhejiang businessmen have created a new world of entrepreneurship, and achieved brilliant achievements that attracted worldwide attention.

In today’s information age, Zhejiang businessmen, with their consistent sensitivity and quickness, are performing a new interpretation of Zhejiang business culture with the brand-new social values and cultural imagination contained in the emerging culture. Zhejiang businessmen pay more attention to the transformation from traditional industrialized economy to modern service-oriented, innovative and digital economy. Centering on the five fields of network economy, high-end manufacturing, bioeconomy, green low-carbon and digital creativity, they focus on the development of ten strategic emerging industries such as information technology, Internet of things, artificial intelligence, high-end equipment manufacturing, new materials, biology, new energy vehicles, new energy, energy conservation and environmental protection and digital creativity, of which the output value of information technology industry exceeded 250 billion yuan in 2020. A number of innovative enterprises such as Alibaba and NetEase have emerged.

#### The Influence of Southern Fujian Business Culture on the Development of Enterprises in Fujian

In Fujian, the song “Working hard can win” is widely known. In a sense, this song portrays Fujian businessmen. Looking back on the history, Fujian businessmen always show spirits of flexibility, risk-taking, perseverance and brotherhood. In southern Fujian, one often hears “work hard when young, or enjoy no reputation in later life.” Influenced by the tradition of doing business, people in southern Fujian think highly of “set up business and be the boss.” Instead of saving money, they like to start up business, taking it the greatest fulfillment (Huang et al., 2012).

The spirit of daring to take risks and fighting makes most people in Fujian wishing to be the “head” and not the “attendant” since childhood (Luo and Peng, 1999). The positive enterprising and pioneering spirit of Min-nan culture has been popular in many enterprises. In the 1990s, it has always been the seller’s market in sports shoes industry. Almost all shoe factories in Fujian have continuous orders, and all shoes they made can be quickly sold in the market. However, Ding Zhizhong, leader of Anta Group, was not satisfied with the current situation. He and his team believed that if they were satisfied with the current situation, they were doomed to be eliminated by the market. Therefore, Anta began to hire sports stars as product spokesmen, which gave Anta the opportunity to enter the brand era dominated by sports marketing and changed from producing only a single product of sports shoes to producing comprehensive sporting goods.

#### The Influence of Guangdong Business Culture on the Development of Enterprises in Pearl River Delta

The basic characteristics of Guangdong business culture are inclusiveness, pragmatism, profit worship and flexibility (Dong and Liu, 2010). This cultural characteristic makes entrepreneurs in the Pearl River Delta have special sensitivity to business and making money in social psychology, public mentality and values. They are good at changing with time and seizing the opportunity in action. Due to its openness and inclusiveness, it can always absorb excellent achievements in real economic activities to promote the development and improvement of commercial civilization. In the early stage of reform and opening up, Guangdong took the lead in practicing the concept of market economy, began a series of ownership reforms, reformed state-owned enterprises and explored the establishment of joint-stock enterprises.

Businessmen in the Pearl River Delta go abroad, actively expand overseas markets and generally adopt the strategy of “trade first and manufacturing follow-up” (Gan and Zheng, 2009). After the trade volume has expanded to a certain scale and accumulated experience in international market operation, businessmen further explore a new path of overseas investment and plant construction. For example, Midea, TCL, Konka, Gree and other enterprises have invested and set up factories overseas, especially in developing countries, becoming the forerunner of overseas expansion of Chinese enterprises. A large number of representative enterprises such as ZTE and Huawei have set a good example. While inheriting pragmatic, accessible and other spiritual culture, the Pearl River Delta business gang has more innovative consciousness and long-term vision.

### Economic Comparison of the Provinces Where the Five Modern Business Gangs Are Located

#### Comparison of the Provinces Where the Five Modern Business Gangs Are Located

By the end of 2019, the basic situation of the provinces where the five modern business gangs are located is shown in Table 1.

**TABLE 1:** General information of the provinces where the five modern business gangs are located (By the end of 2019).

	Shandong	Jiangsu	Zhejiang	Fujian	Guangdong
Area	The total land area of Shandong is 15,712,600 square kilometers.	The total area of Jiangsu is 107,200 square kilometers.	The total area of Zhejiang is 105,500 square meters.	The total land area of Fujian is 121,400 square kilometers.	The total area of Guangdong is 179,700 square kilometers.
Permanent resident population	10,702,100 million people	80,700,000 million people	58,500,000 million people	39,730,000 million people	115,210,000 million people
Education situation	There were 34 graduate schools with 115,000 students, 146 colleges and universities with 2,184,000 students, 391 secondary vocational schools (excluding technical schools) with 730,000 students, and 181 technical schools with 355,000 students.	There were 215,000 graduate students, 142 colleges and universities with 1,874,000 students, and 622,000 students in secondary vocational schools (excluding technical schools).	There were 109 colleges and universities (including independent colleges), and 245 secondary vocational schools (excluding technical schools) with 542,000 students.	There were 58,700 graduate students, 89 colleges and universities with 861,200 students, and 334,800 students in secondary vocational schools (excluding technical schools).	There were 147 colleges and universities in Guangdong. The number of students in graduate education is 154700, and the number of ordinary college students is 240200.
Natural resources	Shandong has abundant mineral resources, taking an important place in China.	Jiangsu is rich in non-ferrous metals, building materials, gypsum-salts, and special non-metallic minerals.	Zhejiang has a wide variety of minerals with alumite reserves ranking first in the world (60%), fluorite reserves second in China.	As one of the important mineralization areas in the Pacific Rim mineralization zone, Fujian is rich in mineral resources.	Guangdong is rich in water resources and has a wide variety of plants and animals. Guangdong is home to rare and non-ferrous metals.

*China Statistical Yearbook.*

#### Comparison of Economic Conditions of the Five Provinces

**FIGURE 2:**
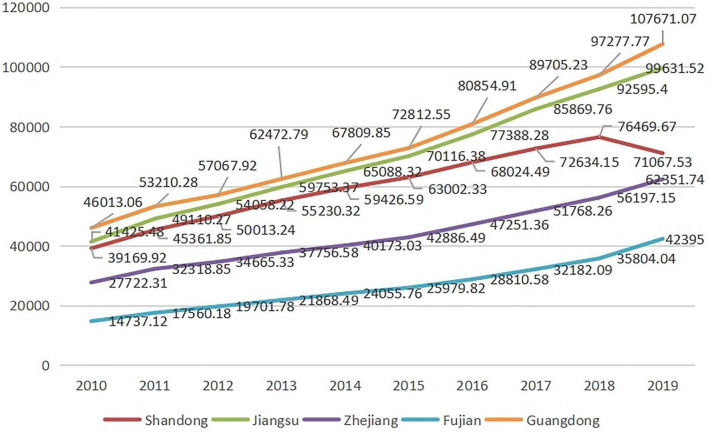
Trends of GDP in the five provinces (2010−2019).

As Table 2 and Figure 2 show, Guangdong has always been the first, followed by Jiangsu, Shandong, Zhejiang and Fujian. The ranking was related to number of enterprises and quantity of labor forces in the provinces. Except for Shandong, where GDP fell slightly in 2019, the GDP of other provinces showed an upward tendency in 2010–2019.

**TABLE 2:** GDP in the five provinces (2010–2019).

Year	Gross domestic product (100 million yuan)				
	Shandong	Jiangsu	Zhejiang	Fujian	Guangdong
2019	71067.53	99631.52	62351.74	42395.00	107671.07
2018	76469.67	92595.40	56197.15	35804.04	97277.77
2017	72634.15	85869.76	51768.26	32182.09	89705.23
2016	68024.49	77388.28	47251.36	28810.58	80854.91
2015	63002.33	70116.38	42886.49	25979.82	72812.55
2014	59426.59	65088.32	40173.03	24055.76	67809.85
2013	55230.32	59753.37	37756.58	21868.49	62472.79
2012	50013.24	54058.22	34665.33	19701.78	57067.92
2011	45361.85	49110.27	32318.85	17560.18	53210.28
2010	39169.92	41425.48	27722.31	14737.12	46013.06

*China Statistical Yearbook.*

#### Proportion of Legal Entities by Industry in the Five Provinces in 2019

**FIGURE 3:**
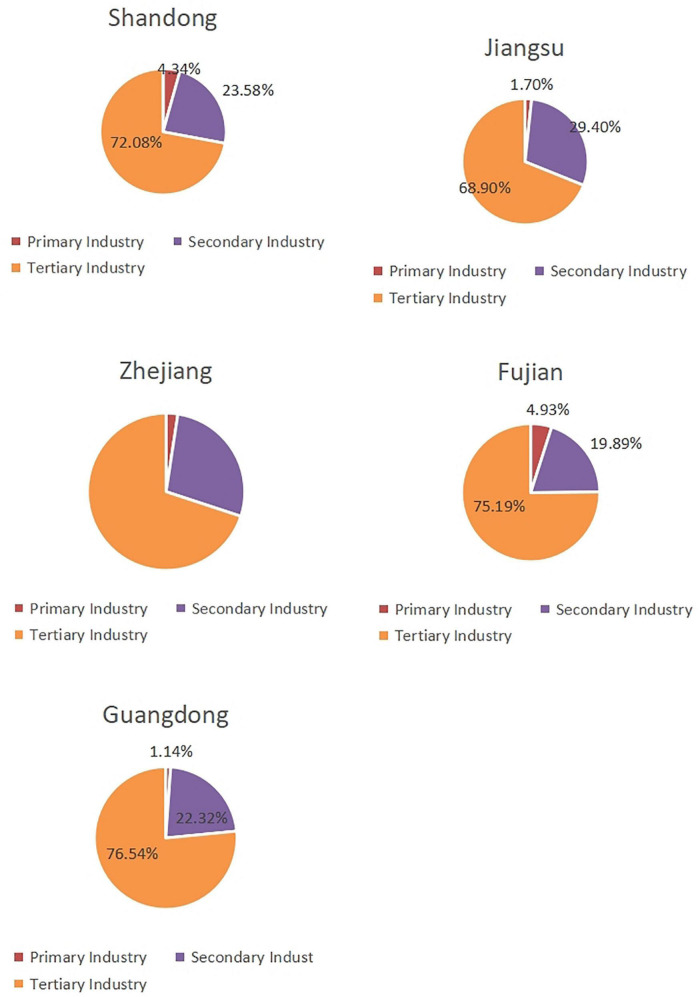
Proportion of legal entity by industry in the five provinces.

According to the proportion of legal entities in the three major industries, the tertiary industry had a dominate position in all five provinces (greater than 60%), while the primary industry accounted for a small proportion (less than 5%), as shown in Figure 3.

Compared with other provinces, the proportion of primary industry in Fujian was highest (4.93%), while that of Guangdong was the lowest (1.14%). As to secondary industry, the proportion in Jiangsu was the highest (29.40%), while that of Fujian was the lowest (19.89%). As to tertiary industry, the proportion in Guangdong was the highest (76.54%), while that of Jiangsu was the lowest (68.90%).

#### GDP by Industry in the Five Provinces in 2019

**FIGURE 4:**
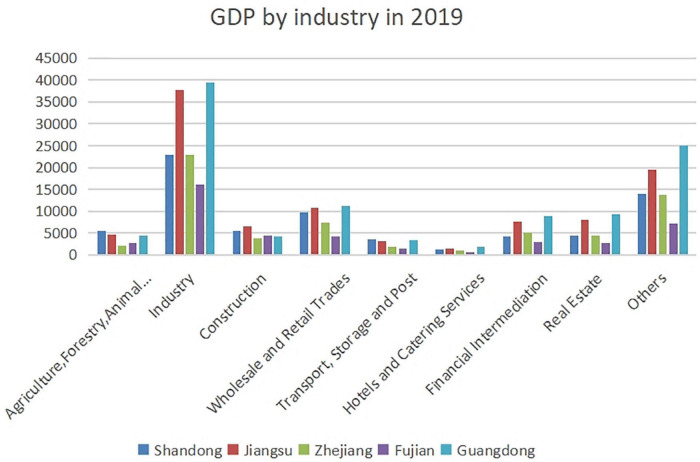
GDP by industry in the five provinces.

As shown in Figure 4, the GDP by industry differed significantly in the five provinces, which was closely related to the development model and industry type of regional economy. Overall, the five provinces had higher gross industrial production, followed by wholesale and retail trades.

The development of agriculture, forestry, animal husbandry and fishery, as well as transport, storage and post, was prominent in Shandong. The industry, financial intermediation, and real estate developed rapidly in Jiangsu. GDP in Zhejiang was greatly influenced by the industry and wholesale and retail trades, while agriculture, forestry, animal husbandry and fishery contributed little to GDP. Fujian had a higher gross industrial production. In Guangdong, GDPs of the industry, wholesale and retail trades, financial intermediation and real estate were relatively higher.

#### Differences in Industrial Added Value in the Five Provinces

Modern industry is divided into primary industry, secondary industry and tertiary industry. This study gathered from China Statistical Yearbook data about added value in different industries in the five provinces (see Table 3). A one-way ANOVA was conducted to analyze the differences in industrial added value in the five provinces, as shown in Tables 4, 5.

**TABLE 3:** Added value by industry in the five provinces in 2015–2019.

Year	Industry	Value added									
		Shandong		Jiangsu		Zhejiang		Fujian		Guangdong	

		Value added(100 million)	%	Value added(100 million)	%	Value added(100 million)	%	Value added(100 million)	%	Value added(100 million)	%
2015	Primary industry	4979.08	7.90%	3986.05	5.68%	1832.91	4.27%	2118.1	8.15%	3345.54	4.59%
	Secondary industry	29485.9	46.80%	32044.45	45.70%	19711.67	45.96%	13064.82	50.29%	32613.54	44.79%
	Tertiary industry	28537.35	45.30%	34085.88	48.61%	21341.91	49.76%	10796.9	41.56%	36853.47	50.61%
	Total	63002.33		70116.38		42886.49		25979.82		72812.55	
2016	Primary industry	4929.13	7.25%	4077.18	5.27%	1965.18	4.16%	2363.22	8.20%	3694.37	4.57%
	Secondary industry	31343.67	46.08%	34619.5	44.73%	21194.61	44.86%	14093.47	48.92%	35109.56	43.42%
	Tertiary industry	31751.69	46.68%	38691.6	50.00%	24091.57	50.99%	12353.89	42.88%	42050.88	52.01%
	Total	68024.49		77388.28		47251.36		28810.58		80854.91	
2017	Primary industry	4832.71	6.65%	4045.16	4.71%	1933.92	3.74%	2215.13	6.88%	3611.44	4.03%
	Secondary industry	32942.84	45.35%	38654.87	45.02%	22232.08	42.95%	15354.29	47.71%	38008.06	42.37%
	Tertiary industry	34858.6	47.99%	43169.73	50.27%	27602.26	53.32%	14612.67	45.41%	48085.73	53.60%
	Total	72634.15		85869.76		51768.26		32182.09		89705.23	
2018	Primary industry	4950.52	6.47%	4141.72	4.47%	1967.01	3.50%	2379.82	6.65%	3831.44	3.94%
	Secondary industry	33641.72	43.99%	41248.52	44.55%	23505.88	41.83%	17232.36	48.13%	40695.15	41.83%
	Tertiary industry	37877.43	49.53%	47205.16	50.98%	30724.26	54.67%	16191.86	45.22%	52751.18	54.23%
	Total	76469.67		92595.4		56197.15		35804.04		97277.77	
2019	Primary industry	5116.44	7.20%	4296.28	4.31%	2097.38	3.36%	2596.23	6.12%	4351.26	4.04%
	Secondary industry	28310.92	39.84%	44270.51	44.43%	26566.6	42.61%	20581.74	48.55%	43546.43	40.44%
	Tertiary industry	37640.17	52.96%	51064.73	51.25%	33687.76	54.03%	19217.03	45.33%	59773.38	55.51%
	Total	71067.53		99631.52		62351.74		42395		107671.07	

*China Statistical Yearbook.*

**TABLE 4:** Results of the one-way ANOVA for industrial added value.

	Sum of squares	df	Mean square	F	Sig.
Between groups	11118754208.169	4	2779688552.042	4.584	0.002
Within groups	57611137793.470	95	606433029.405		
Total	68729892001.640	99			

**TABLE 5:** Comparison of differences in industrial added value in the five provinces.

Groups	N	Subsets (alpha = 0.05)	
		1	2
Added value in Fujian	20	16517.1530	
Added value in Zhejiang	20	26045.5000	26045.5000
Added value in Shandong	20		35119.8170
Added value in Jiangsu	20		42560.1340
Added value in Guangdong	20		44832.1480
Significance		0.224	0.082

A one-way ANOVA was conducted using SPSS software to analyze the differences in economic growth in the five provinces. As can be seen from Table 4, the *p*-value was less than 0.05, indicating the differences were significant. Then a pairwise comparison analysis was performed to reveal the differences in economic growth in the five provinces, as illustrated in Table 5.

From Table 5, it can be seen that significance value in Subset 1 is 0.224 (> 0.05), indicating that there is no significant difference in industrial added value between Fujian and Zhejiang. The significance value in Subset 2 (0.082) shows that there is no significant difference between Zhejiang and Shandong, Jiangsu, Guangdong. This further indicates that the difference in industrial added value between Zhejiang and Fujian is not significant, while the difference between Fujian and Shandong, Jiangsu, Guangdong is significant. Cultures of Fujian and Zhejiang show a greater similarity, and Zhejiang culture has much in common with cultures of Shandong, Jiangsu and Guangdong. This suggests that Zhejiang culture takes in elements of cultures in other regions, presenting a diversified characteristic. This diversified culture offers supports for development of Zhejiang business gang. Fujian culture shows great difference from cultures in Shandong, Jiangsu and Guangdong, and is rather distinct.

## Results

The data presented above, including GDP of the five provinces, proportion of legal entities by industry, GDP by industry in these regions and added value by industry, shows that there are many differences in regional economic development, including the growing rate of regional GDP, the proportion of legal entities in the three major industries, and the regional GDP by industry.

As can be seen from the above analysis, there is a crucial connection between culture and economy, and regional culture with local characteristics exerts a profound impact on the development of regional economy (Ying and Su, 2018). Regional culture can promote or restrict the development of regional economy (Perroux, 1950). When regional culture penetrates into the regional economy through the behavior of entrepreneurs, it can form a characteristic regional economic model. Through case and data analysis, it is found that as a bridge between regional culture and regional economy, China’s modern business gangs use their relationship networks to carry out exchanges, cooperation and mutual assistance, and enhance their sense of identity and belonging. They have strong cohesion, which is conducive to the formation of industrial clusters and the win-win development of business gangs and regional economy.

## Discussion and Conclusion

### Discussion

#### Regional Culture Affects Entrepreneurs’ Psychological Characteristics and Startup Behaviors

Cultural beliefs influence main bodies’ attitude toward new things and new technologies, thus influencing the transformation and sharing of technologies (Horak and Arya, 2020). As to the enterprises, their development depends on the entrepreneurs, and the regional cultural beliefs play an important role in the cultivation, formation and development of entrepreneurship (Kaasa et al., 2014). Any individual has certain psychological characteristics and behavioral pattern. As this study shows, under the influence of different regional cultures, there are significant differences in the psychological characteristics and startup behaviors of business entrepreneurs.

Through the comparative analysis, it is found that under the influence of Confucian culture, Shandong business gang has formed the psychological characteristics of advocating benevolence, propriety and hard-working, paying attention to pragmatism and pursuing ideals; Influenced by the water culture in southern Jiangsu, the Southern Jiangsu business gang has formed the psychological characteristics of advocating industry and practical application. Entrepreneurs operate carefully and are good at management; Under the influence of eastern Zhejiang culture, Zhejiang business gang has formed the psychological characteristics of diligence, pragmatism and bravery, and is good at seizing opportunities and creating innovations; Influenced by the marine culture of Southern Fujian, the Southern Fujian business gang has formed the psychological characteristics of daring to take risks and struggle, and pays attention to the development of market economy under the background of internationalization; influenced by the culture of southern Guangdong, the Pearl River Delta business gang has formed the psychological characteristics of freedom, openness and adaptiveness, and has the courage to be the developer and leader of the global market.

#### Entrepreneurs’ Psychological Characteristics and Startup Behaviors Affect the Development of Enterprises and Business Gangs

As an informal institution, culture has great power and can gradually influence the main bodies of regional development, which in turn influence all aspects of social and economic development. Thus, culture constitutes the background and context of regional economic development (Mattli et al., 2019). By influencing entrepreneurs’ psychological characteristics and startup behaviors, regional culture makes enterprises participate in regional economic cycle and become an important factor for sustainable regional economic development and regional characteristics.

Influenced by traditional culture, Shandong business gangs pay attention to close contact with the government. Agriculture, forestry, animal husbandry and fishery, transportation, storage and postal industry have developed prominently, and there are relatively many large state-owned enterprises; Southern Jiangsu business gangs pay attention to the effective organization and overall operation. They have developed rapidly in industry, finance and real estate, and a number of influential collective ownership enterprises have emerged; Zhejiang Business gangs pay attention to the development of processing and manufacturing industry, Internet and trade circulation service industry, and private enterprises and industrial clusters are highly developed; Southern Fujian business gangs form the characteristics of combining domestic and foreign trade, with obvious internationalization and marketization characteristics. Southern Fujian businessmen are most widely distributed in the world; Most of the Pearl River Delta business gangs are in industry, wholesale and retail and finance. They have developed a number of export-oriented processing and manufacturing enterprises and become the forerunner of the overseas expansion of the Pearl River Delta business gangs.

### Conclusion

In summary, the hypothesis put forward in this paper is verified through comparative analysis. Culture, as an informal system, has an impact on entrepreneurs’ psychological characteristics, startup behaviors, enterprise development and regional economy. Due to different regional cultures, distinctive psychological characteristics and startup behaviors will be formed, which will result in differences of enterprises in industrial selection, business philosophy, cooperation mode and market strategy, and thus affect regional economic vitality, regional innovation, regional comprehensive strength and regional industrial distribution.

With the improvement of market economy, the regional economic development with modern business gang as the main body is increasingly linked with regional culture (Beugelsdijk et al., 2019). On the one hand, the development of modern business gang is the material foundation of culture prosperity. On the other hand, regional culture has a profound impact on the development of modern business gang. The mutual promotion of regional culture and regional business can promote the long-term development of regional economy. Through the above research, this paper puts forward the following suggestions.

#### Fully Tap the Connotation Value of Regional Culture in Economic Development

Actively tap the rich connotation of regional culture, make use of regional cultural resources and mainstream publicity channels, carry forward the values and world outlook advocated by regional culture, and give play to its positive and unique role in economic and social development and the improvement of people’s livelihood. Guided by the market and driven by culture, give play to regional cultural advantages through cultural guidance, typical demonstration and other ways. Promote the comprehensive, coordinated and sustainable development of modern business gangs and regional economy.

#### Attach Great Importance to the Positive Role of Regional Culture in the Growth of Entrepreneurs

Entrepreneurs are important commanders and coordinators to promote the aggregation of various economic development factors. The psychological and behavioral characteristics of entrepreneurs play an increasingly important role in the operation and development of enterprises. Culture plays a subtle role in the growth of entrepreneurs. We should give full play to the influence of advanced regional culture on the cluster growth of business entrepreneurs, enhance the competitive advantage of business gangs, and promote regional economic growth.

#### Give Full Play to the Cluster Effect of Business Gangs on Regional Economic Development

We should make use of the cluster effect of modern business gangs to make it easier for entrepreneurs to communicate and cooperate in the cluster of regional business gangs, promote benign competition and win-win development among enterprises. Thus business gangs have a stronger competitive advantage than a single enterprise, so as to continuously improve the comprehensive competitiveness in the cluster. Form a new pattern of regional economic development with obvious agglomeration effect and distinctive industrial characteristics.

## Research Limitations

The limitation of this study is that the data mainly comes from the National Statistical Yearbook, which has strong data authority, but the source channels and the number of samples are limited. Therefore, the comprehensiveness and diversity of data can be further improved in the future. We can also carry out case studies for more regional business gangs to further improve the validity of the article, so as to make greater contributions to the research on the relationship between regional culture, regional business gangs and regional economic development.

## Data Availability Statement

The original contributions presented in the study are included in the article/supplementary material, further inquiries can be directed to the corresponding author/s.

## Ethics Statement

Ethical review and approval was not required for this study on human participants in accordance with the local legislation and institutional requirements. Written informed consent for participation was not required for this study in accordance with the national legislation and the institutional requirements.

## Author Contributions

JL determined the research theme, research framework, data analysis method, and was responsible for the finalization of the manuscript. WL was responsible for literature collation, data collection, and draft writing. YS was responsible for data analysis and result discussion. All authors contributed to the study and approved the submitted version.

## Conflict of Interest

The authors declare that the research was conducted in the absence of any commercial or financial relationships that could be construed as a potential conflict of interest.

## Publisher’s Note

All claims expressed in this article are solely those of the authors and do not necessarily represent those of their affiliated organizations, or those of the publisher, the editors and the reviewers. Any product that may be evaluated in this article, or claim that may be made by its manufacturer, is not guaranteed or endorsed by the publisher.

## References

[B1] Ajzen I. (1991). The theory of planned behavior. *Organ. Behav. Hum.* 50 179–211. 10.1016/0749-5978(91)90020-T

[B2] Baldwin R. (1999). Agglomeration and endogenous capital. *Eur. Econ. Rev.* 43 253–280. 10.1016/S0014-2921(98)00067-1

[B3] Baldwin R. Martin P. Ottaviano G. (2001). Global income divergence, trade and industrialization: the geography of growth take-off. *J. Econ. Dev.* 66 5–37. 10.2139/ssrn.92169

[B4] Beugelsdijk S. Klasing M. J. Milionis P. (2019). Value diversity and regional economic development. *Scand. J. Econ.* 121 153–181. 10.3390/su122310014

[B5] Chen W. L. (2004). Review of new business gangs in China. *Bus. China* 8:48.

[B6] Chen X. Han T. X. (2008). The cultural elements and economic increase. *Econ. Theor. Bus. Manag.* 9 12–18. 10.3969/j.issn.1000-596X.2008.09.002

[B7] Dong K. Liu Y. (2010). Cross-cultural management in China. *Cross Cult. Manag.* 17 223–243. 10.1108/13527601011068333

[B8] Dong L. Glaister K. W. (2007). National and corporate culture differences in international strategic alliances: perceptions of chinese partners. *Asia Pac. J. Manag.* 24 191–205. 10.1007/s10490-006-9010-7

[B9] Feng S. S. Yang Z. P. Feng J. (2009). Economic promotion of wu merchants and economic rise of central part of zhejiang province. *Bus. Econ.* 23 1–12. 10.3969/j.issn.1009-6043.2009.23.001

[B10] Gan C. H. Zheng R. G. (2009). An empirical study on change of industrial structure and productivity growth since the reform and opening-up——a test for the structure-bonus hypotheses from 1978 to 2007 in china. *China Indu. Econ.* 31 281–286. 10.1007/978-3-642-02298-2_32

[B11] Gao F. (2012). New business gangs in China. *Shanghai Enterp.* 5 34–35.

[B12] Ge W. F. (2008). On the independent innovation of Zhejiang businessmen from the perspective of modern business gangs. *J. Fujian Prov. Com. Party Sch. CPC* 3 76–79.

[B13] Horak S. Arya B. (2020). Cultural context and cross-country behavioral differences in group decision-making. *Int. Stud. Manag. Organ.* 50 153–173. 10.1080/00208825.2020.1758423

[B14] Huang J. P. Wu Y. Y. Li W. H. (2012). Application of Fujian businessmen’s spirit to the moral education in Fujian-Taiwan cooperative education programs. *Youth* 8 148–149.

[B15] Isard W. (1949). The general theory of local and space-economy. *Q. J. Econ.* 62 476–506. 10.2307/1882135

[B16] Kaasa A. Vadi M. Varblane U. (2014). Regional cultural differences within European countries: evidence from multi-country surveys. *Manag. Int. Rev.* 54 120–125. 10.1007/s11575-014-0223-6

[B17] Koslowski P. (2010). Elements of a philosophy of management and organization. *Stud. Econ. Ethics Philos.* 2 195–210. 10.1109/PAIEE.1907.6741820

[B18] Krugman P. R. (1991). Increasing returns and economic geography. *J. Pol. Econ.* 99 483–499. 10.2307/2937739

[B19] Luo P. Peng M. W. (1999). Learning to compete in a transition economy: experience, environment, and performance. *J. Int. Bus. Stud.* 30 269–295. 10.1057/palgrave.jibs.8490070

[B20] Lv M. Y. You M. M. (2014). An analysis of Changing trends in the manufacturing industry structure of China’s three major economic circles: an empirical study base on the Surrounding Bohai Sea, the Yangzi River Delta and the Pearl River Delta. *Indu. Econ. Rev.* 5 27–39. 10.14007/j.cnki.cjpl.2014.03.005

[B21] Marshall A. (1890). *Principles of Economics.* Dongcheng: Huaxia Press, 232–355.

[B22] Mattli R. Wieser S. Probst-Hensch N. Schmidt-Trucksäss A. Schwenkglenks M. (2019). Physical inactivity caused economic burden depends on regional cultural differences. *Scand. J. Med. Sci. Sport* 29 95–104. 10.1111/sms.13311 30260508

[B23] Muthukrishna M. Bell A. V. Henrich J. Curtin C. M. Gedranovich A. McInerney J. (2020). Beyond western, educated, industrial, rich, and democratic (WEIRD) psychology: measuring and mapping scales of cultural and psychological distance. *Psychol. Sci.* 31 678–701. 10.1177/0956797620916782 32437234 PMC7357184

[B24] Nong C. G. (2016). On the development of regional economy from the perspective of regional culture. *W. R.* 6 191–192. 10.16631/j.cnki.cn15-1331/p.2016.06.076

[B25] North D. C. (1955). Location theory and regional economic growth. *J. Poli. Econ.* 63 243–243. 10.1086/257668

[B26] Perroux F. (1950). Economic space: theory and applications. *Q. J. Econ.* 65 89–104. 10.2307/1881960

[B27] Prasetyo P. Setyadharma A. Kistanti N. R. (2020). Potential of new institutional economics for rural community development. *SHS Web. Conf.* 86:01015. 10.1051/shsconf/20208601015

[B28] Quek M. T. Storm C. L. (2012). Chinese values in supervisory discourse: implications for culturally sensitive practices. *COFT* 34 44–56. 10.1007/s10591-011-9172-4

[B29] Wang D. (2018). The relationship between regional culture and regional economic growth. *China Econ.* 8 150–152.

[B30] Woodworth R. (1916). *Dynamic Psychology.* Haidian: People’s University of China Press, 25–121.

[B31] Wu X. P. Gao B. (2007). Culture, entrepreneurship and economic growth: based on the previous literatures and experience observation. *J. Shanxi Univ. Fina. Econ.* 6 74–80.

[B32] Xia L. L. (2000). A study of the culture influence on the regional economy. *Hum. Geogr.* 15 55–75.

[B33] Yerznkyan B. Gassner L. (2018). Cultural and institutional differences at the national and regional levels. *Int. J. Econ. Fin. Manag. Sci.* 6 80–86. 10.11648/j.ijefm.20180604.11

[B34] Ying L. Su W. (2018). Regional difference of innovation efficiency of cultural and creative enterprises in China based on DEA approach. *J. Dis. Math. Sci. Cryptogr.* 21 583–587. 10.1080/09720529.2018.1453662

[B35] Yu J. H. Nan L. J. (2013). On the positive or negative correlation between culture and economy. *Admi. Tri.* 20 84–87. 10.3969/j.issn.1005-460X.2013.06.017

[B36] Zhang W. C. (2010). Can an ancient culture revive? *Bus. W.* 24:28.

[B37] Zhang Y. L. Tai X. D. (2015). The impact of regional culture on market economic environment. *Rev. Econ. Manag.* 31 132–137. 10.13962/j.cnki.37-1486/f.2015.02.019

[B38] Zhejiang Business website (2006). Available online at: http://zxyzt.zjol.com.cn/zxyw/202012/t20201230_21894490.shtml (accessed December 30, 2020).

